# Comparative Effectiveness of Bariatric Surgery Versus GLP‐1 Receptor Agonists in Reducing the Risk of New‐Onset of NASH: A Retrospective Multinational Cohort Study From North America and Europe

**DOI:** 10.1002/edm2.70075

**Published:** 2025-07-12

**Authors:** Abdallah Hussein, Ameer Awashra, Islam Rajab, Mohammad Bdair, Dawoud Hamdan, Ahmad Nouri, Elaf Khatib, Ghiras Khatib, Nyan Latt

**Affiliations:** ^1^ Internal Medicine Department Virtua Health Inc. Voorhees New Jersey USA; ^2^ Department of Medicine An Najah National University Nablus Palestine; ^3^ Internal Medicine Department St. Joseph's University Medical Center Paterson New Jersey USA; ^4^ College of Health Professions, Thomas Jefferson University Philadelphia Pennsylvania USA; ^5^ School of Public Health, Drexel University Philadelphia Pennsylvania USA; ^6^ Gastroenterology and Hepatology Virtua Health Inc. Voorhees New Jersey USA

**Keywords:** bariatric surgery, GLP‐1 receptor agonists, hepatocellular carcinoma, liver cirrhosis, metabolic dysfunction, nonalcoholic steatohepatitis (NASH), obesity, propensity score matching, real‐world data, retrospective cohort study

## Abstract

**Background:**

Nonalcoholic steatohepatitis (NASH) is a severe form of nonalcoholic fatty liver disease (NAFLD) that can progress to cirrhosis and hepatocellular carcinoma (HCC). Obesity is a major risk factor for NASH, and metabolic interventions such as bariatric surgery (BS) and glucagon‐like peptide‐1 receptor agonists (GLP‐1 RAs) have been explored for their impact on liver‐related outcomes. This study evaluates the comparative effectiveness of BS and GLP‐1 RAs in reducing the incidence of new‐onset NASH and related hepatic complications.

**Methods:**

This was a large, population‐based, retrospective cohort using data from the TriNetX platform. Adult patients with a body mass index (BMI, of 35 or greater and without a history of NAFLD/NASH (without cirrhosis) who underwent BS versus GLP‐1RA between January 1, 2014 and December 31, 2019, were included. Patients in the BS group were matched with patients in the GLP‐1RA group according to age, demographics, comorbidities and medication by using 1:1 propensity matching.

**Results:**

Among 180,022 eligible adults, 143,404 underwent BS, while 36,618 received GLP‐1 RA therapy. Following propensity score matching, 33,594 patients in the BS group (mean age 49.1 ± 13.2 years; 72.73% female) were matched to an equal number of individuals in the GLP‐1 RA group (mean age 48.9 ± 14.0 years; 72.41% female). Compared to those receiving GLP‐1 RA therapy, patients who underwent BS had a significantly lower risk of HCC (HR, 0.304; 95% CI, 0.099–0.931), which showed the strongest protective effect, followed by a substantial reduction in NASH (HR, 0.509; 95% CI, 0.469–0.551). The reduction in liver cirrhosis risk was not statistically significant (HR, 0.865; 95% CI, 0.696–1.075). These associations remained across follow‐up periods of 1, 3, 5 and 7 years.

**Conclusions:**

These findings suggest that BS was significantly associated with lower risk of new onset of NASH/NAFLD.

## Graphical abstract

A real‐world study compares bariatric surgery and GLP‐1 receptor agonists in reducing NASH‐related complications. Bariatric surgery showed superior effectiveness in hepatic outcomes, but higher all‐cause mortality warrants further investigation.

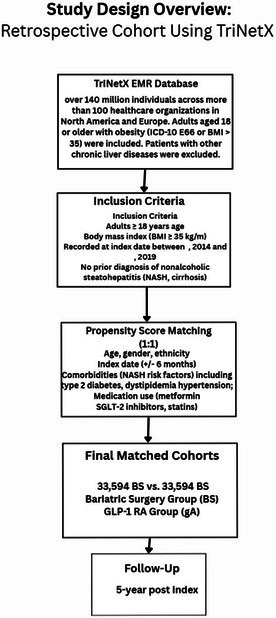

## 1 Introduction

Obesity has emerged as a significant global health challenge, driving the increasing burden of metabolic disorders, including nonalcoholic fatty liver disease (NAFLD) and its progressive form, nonalcoholic steatohepatitis (NASH) [1, 2]. NASH is characterised by hepatic steatosis, inflammation and fibrosis, which can ultimately lead to cirrhosis, hepatocellular carcinoma (HCC) and liver‐related mortality [3]. Given the close association between obesity, insulin resistance and liver disease progression, interventions targeting weight reduction and metabolic improvement have become central to NASH management [4].

Despite the growing understanding of NASH pathogenesis, effective therapeutic options remain limited. The recent FDA approval of resmetirom marks a breakthrough in NASH pharmacotherapy, yet lifestyle interventions, metabolic surgery and other pharmacological treatments continue to be explored as viable strategies [5]. Among these, glucagon‐like peptide‐1 receptor agonists (GLP‐1 RAs) and bariatric surgery (BS) have shown promising effects on weight loss, insulin sensitivity and hepatic fat reduction, positioning them as potential interventions for mitigating NASH progression [6].

GLP‐1 RAs, initially developed for type 2 diabetes management, have demonstrated hepatoprotective effects through mechanisms such as appetite suppression, improved glucose homeostasis and direct anti‐inflammatory and antifibrotic properties in the liver [6]. Clinical studies have highlighted their ability to reduce hepatic steatosis and improve histological markers of NASH. Similarly, bariatric surgery, particularly Roux‐en‐Y gastric bypass and sleeve gastrectomy, has been associated with significant weight loss, resolution of metabolic comorbidities and improvement in liver histology. Long‐term data suggest that BS may lead to the regression of fibrosis and even the resolution of NASH in a substantial proportion of patients [7].

While both GLP‐1 RAs and BS offer metabolic benefits, their comparative effectiveness in preventing new‐onset NASH and cirrhosis remains unclear. Understanding their relative impact on liver‐related outcomes is crucial for guiding clinical decision‐making in obesity management. To address this gap, we conducted a retrospective cohort study evaluating the risk of new‐onset NASH and liver cirrhosis in patients treated with GLP‐1 RAs versus those who underwent bariatric surgery. Our study aims to provide insights into the long‐term hepatic outcomes associated with these interventions, ultimately contributing to the optimisation of NASH prevention strategies in high‐risk populations.

## 2 Methods

We performed a retrospective cohort study using the TriNetX platform. The TriNetX research platform is a global collaborative network providing access to real‐time anonymized electronic medical records. TriNetX has data usage and publication agreements in place with all health care organisations (HCOs). The TriNetX Global Collaborative network comprises over 140 million individuals across over 100 health care organisations (HCOs), including primary, secondary and tertiary units in North America and Europe. Data contained within the network includes demographics, diagnoses, procedures, medications and health care utilisation. We conformed to Strengthening the Reporting of Observational Studies in Epidemiology (STROBE) guidelines [8]. The data used in this study was collected on 25 February 2025.

### 2.1 Primary Cohorts

We identified all adults, aged 18 or over, with obesity defined by the presence of International Classification of Diseases 10th revision (ICD‐10) codes E66 or BMI > 35. We excluded individuals with other causes of chronic liver disease (shown in Table S1). Two groups were created: (1) Patients who underwent bariatric surgery and (2) GLP1‐RA use. GLP1‐RA was defined as Semaglutide and liraglutide, used for at least 1 year. The index event for both cohort groups was defined at one day after the intervention. In addition, we performed an analysis of people who had diabetes type 2 or hyperlipidemia or both as a subgroup.

**FIGURE 1:**
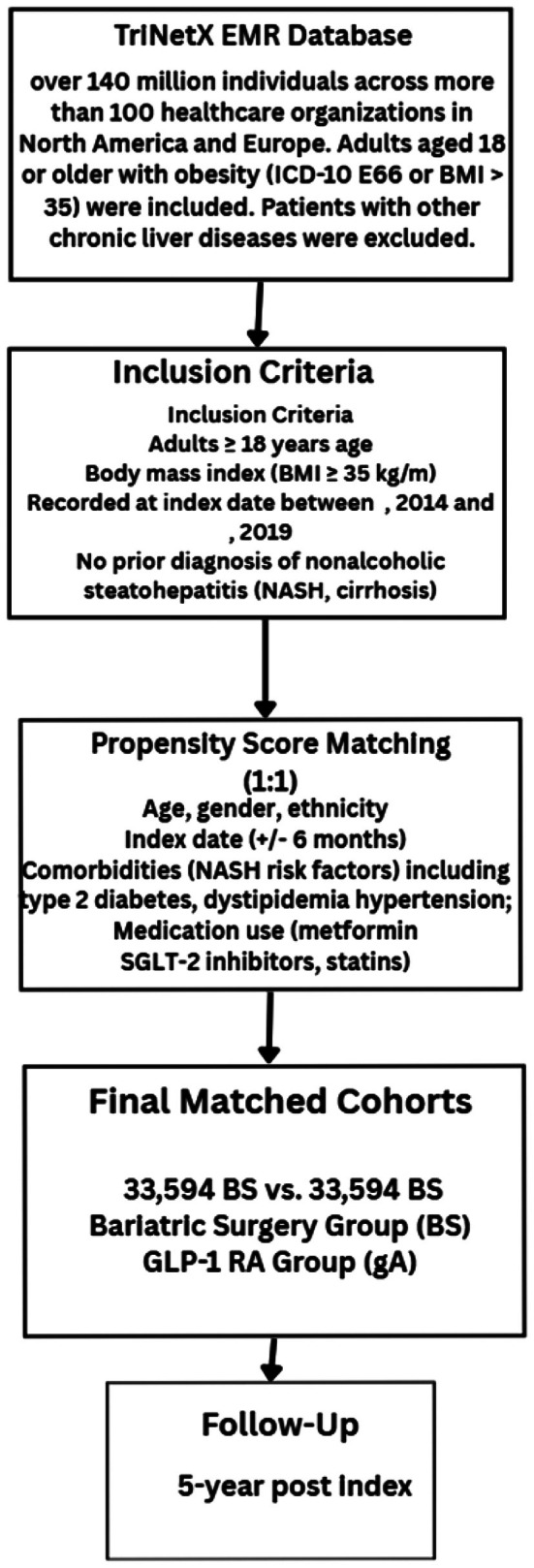
Study design overview of the retrospective cohort using TriNetX platform, showing inclusion criteria, exposure groups, matching method and follow‐up outcomes.

Groups were propensity score matched (PSM) for age at index event, gender, ethnicity, presence of type 2 diabetes (T2D) (ICD‐10 E11), obesity, all neoplasms (ICD‐10 C00‐D49), ischemic heart diseases (ICD‐10 I20‐25). The primary outcome was the incidence of new NASH (ICD‐10 K75.81). Secondary outcomes included incidence of liver cirrhosis (ICD‐10 k74.60), HCC (ICD‐10 C22) and all‐cause mortality. Individuals with a history of an outcome of interest were excluded from prospective analysis of that respective outcome only. Individuals were followed up for 5 years post index event. Participants that died during the study period or who were lost to follow‐up (e.g., moving to another HCO not included within the network) were censored at that time point. Sensitivity analysis was conducted for 5‐year exposure, with outcomes analysed after exclusion outcomes within 1 year or 2 years after the index event. A visual overview of the study design, including cohort selection, matching and follow‐up, is provided in Figure 1.

### 2.2 Statistical Analysis

Due to the nature of the data source, this dataset may face some data quality challenges of EMRs such as incomplete or inaccurate data entries, underreporting of certain conditions, limited granularity and exclusion of data not integrated into the HCO's EMR. Nevertheless, TriNetX employs data validation processes to ensure the accuracy and reliability of its data. These processes include regular data quality checks to identify and correct discrepancies, validation against external benchmarks to ensure consistency and accuracy and collaboration with data contributors to resolve any identified issues and improve data quality continuously. Statistical analysis is conducted within the TriNetX platform using the R survival package as a backbone. Groups were 1:1 propensity score matched (PSM). We used greedy nearest neighbour matching with a calliper of 0.1 pooled standard deviations. The proportional hazard assumption was tested using the generalised Schoenfeld approach built into the platform. Hazard ratios alongside 95% CI and *p* values are reported for the prospective analysis and mean and standard deviation for baseline characteristics. Kaplan–Meier curves were calculated for survival probability. If the last data entry (outcomes of interest, date of death, end of data collection or loss to follow up) in the patient's record was in the time window for analysis, the patient was censored on the day after the last fact in their record. We calculated the *E*‐value for each outcome of interest. The *E*‐value is defined as the minimum strength of association, on the risk ratio scale that an unmeasured confounder would need to have with both treatment and outcome to fully explain away a specific treatment–outcome association. There is no threshold of significance for the *E*‐value and it should be interpreted in context with the size of the HR. Variables with strictly standardised mean difference (SSMD) < 0.1 are well matched between groups. Statistical significance is set at the 5% level.

### 2.3 Result

Among 180,022 patients with obesity, 143,404 underwent BS group, while 36,618 received GLP‐1 RAtherapy. After applying propensity score matching (PSM), 33,594 patients were included in each cohort. In the matched population, the mean age was 49.1 ± 13.2 years in the BS group and 48.9 ± 14.0 years in the GLP‐1 RA group, with female patients comprising 72.73% (*n* = 24,433) and 72.41% (*n* = 24,326) of each group, respectively. Post‐matching balance was achieved across baseline characteristics, with standardised mean differences (SMDs) below 0.1, indicating a high degree of comparability (Table 1). Some residual imbalances persisted but remained within acceptable thresholds (SMD < 0.25).

**TABLE 1:** Propensity score matching and baseline characteristics.

	Before matching			After matching		
	Bariatric surgery (no. 143,404)	GLP1‐RA (no. 36,618)	Std. diff.	Bariatric surgery (no. 33,594)	GLP1‐RA (no. 33,594)	Std. diff.
Age at index (Mean ± SD)	46.4 ± 12.8	49.7 ± 14	0.2472	49.1 ± 13.2	48.9 ± 14	0.0149
Female	114,474 (79.83%)	25,788 (70.44%)	0.2188	24,433 (72.73%)	24,326 (72.41%)	0.0071
White	94,812 (66.12%)	22,538 (61.55%)	0.0951	20,623 (61.39%)	20,766 (61.81%)	0.0088
Diagnosis						
Diseases of digestive system	65,573 (45.73%)	11,723 (32.01%)	0.2841	11,909 (35.45%)	11,339 (33.75%)	0.0357
Metabolic disorders^a^	36,512 (25.46%)	11,445 (31.26%)	0.1288	10,636 (31.66%)	10,246 (30.50%)	0.0251
Hypertension	45,185 (31.51%)	10,239 (27.96%)	0.0777	10,049 (29.91%)	9561 (28.46%)	0.0320
Neoplasms	19,475 (13.58%)	6199 (16.93%)	0.0932	5884 (17.52%)	5637 (16.78%)	0.0195
Chronic lower respiratory disease	22,181 (15.47%)	5222 (14.26%)	0.0339	5275 (15.70%)	4958 (14.76%)	0.0263
Heart failure	2955 (2.06%)	946 (2.58%)	0.0347	875 (2.61%)	835 (2.49%)	0.0076
Medications						
Antiemetics	48,221 (33.66%)	10,310 (28.18%)	0.1186	10,132 (30.16%)	9674 (28.80%)	0.0299
Antacids	28,515 (19.88%)	9911 (27.07%)	0.1700	8722 (25.96%)	8447 (25.14%)	0.0188
Laxatives	33,298 (23.22%)	9141 (24.96%)	0.0408	8687 (25.86%)	8224 (24.81%)	0.0318
Antilipemic agents	13,767 (9.60%)	10,275 (28.06%)	0.4859	7590 (22.59%)	7578 (22.56%)	0.0009
Beta blockers	24,983 (17.42%)	8688 (23.73%)	0.1564	7404 (22.04%)	7332 (21.83%)	0.0052
Labs						
Albumin (g/dL)	4.01 ± 0.445 (50.65%)	4.10 ± 0.408 (52.68%)	0.2063	4.03 ± 0.421 (53.27%)	4.10 ± 0.407 (52.19%)	0.1602
Triglycerides (mg/dL)	120 ± 70.8 (35.82%)	141 ± 116 (46.16%)	0.2153	122 ± 74.5 (46.31%)	138 ± 108 (45.04%)	0.0256
Cholesterol (mg/dL)	180 ± 40.2 (35.85%)	181 ± 47.5 (45.82%)	0.0228	180 ± 41.2 (46.02%)	182 ± 47.1 (44.74%)	0.0413
Haemoglobin A1c (%)	5.63 ± 1.16 (31.87%)	6.18 ± 1.81 (42.01%)	0.3596	5.66 ± 1.16 (41.26%)	6.08 ± 1.76 (40.77%)	0.2848
INR	1.08 ± 0.387 (27.61%)	1.13 ± 0.474 (17.47%)	0.2444	1.12 ± 0.536 (18.76%)	1.13 ± 0.478 (18.33%)	0.0157

Abbreviations: GLP1‐RA glucagon‐like peptide‐1 receptor agonists; SD, standard deviation.

^a^ Metabolic disorder category comprises diagnoses based on ICD‐10 codes, including type 2 diabetes, dyslipidaemia, thyroid disorders and metabolic syndrome.

### 2.4 Patient Characteristics

Table 1 describes patient demographics, baseline comorbidities, laboratory parameters and medications in both groups; most variables were similar in both groups (SMD, < 0.1). However, there were some residual differences: patients who underwent BS, compared with those who did not, had a higher baseline BMI (mean [SD], 40.4 [8.52] vs. 38.5 [7.5]; SMD, 0.2330), whereas the non‐BS group had a higher haemoglobin A_1c_ than the BS group (mean [SD], 6.08% [1.76%] vs. 5.66% [1.16%]; SMD, 0.2848). Patients in the non‐BS group had higher mean values of alanine aminotransferases than those in the BS group (26.8 [31] U/L vs. 25.4 [20.7] U/L; SMD 0.0517), whereas other values for the liver chemistries were similar. Medication use was similar in both groups.

### 2.5 Outcome

Mean (SD) follow‐up was 3.7 (1.78) years for the BS group and 3.68 (1.76) years for the GLP1‐RA group. The BS group, compared with the non‐BS group, had significantly lower risk of new‐onset of NASH (HR, 0.509; 95% CI, 0.469–0.551), Liver Cirrhosis HR (HR 0.865, 95% CI, 0.696–1.075), Hepatocellular carcinoma HCC (HR,0.304; 95% CI, 0.099–0.931) (Table 2).

**TABLE 2:** Summary of clinical events comparing bariatric surgery and GLP‐1RA over 5 years post‐matching.

	Outcome	Bariatric surgery (*n* = 33,594)	GLP‐1 RA only (*n* = 33,594)	Hazard ratio (HR)	95% Confidence interval (CI)	*p*	*E*
Five years	NASH	914	1750	0.509	(0.469, 0.551)	< 0.0001	1.165
	Liver cirrhosis	153	175	0.865	(0.696, 1.075)	0.5685	1.2067
	HCC	≤ 10	13	0.304	(0.099, 0.931)	0.0271	0.7639
	All‐cause mortality	1335	1134	1.173	(1.083, 1.269)	≤ 0.0001	1.6234

Abbreviations: GLP‐1RA, glucagon‐like peptide‐1 receptor agonists; HCC, hepatocellular carcinoma; NASH, nonalcoholic steatohepatitis.

**FIGURE 2:**
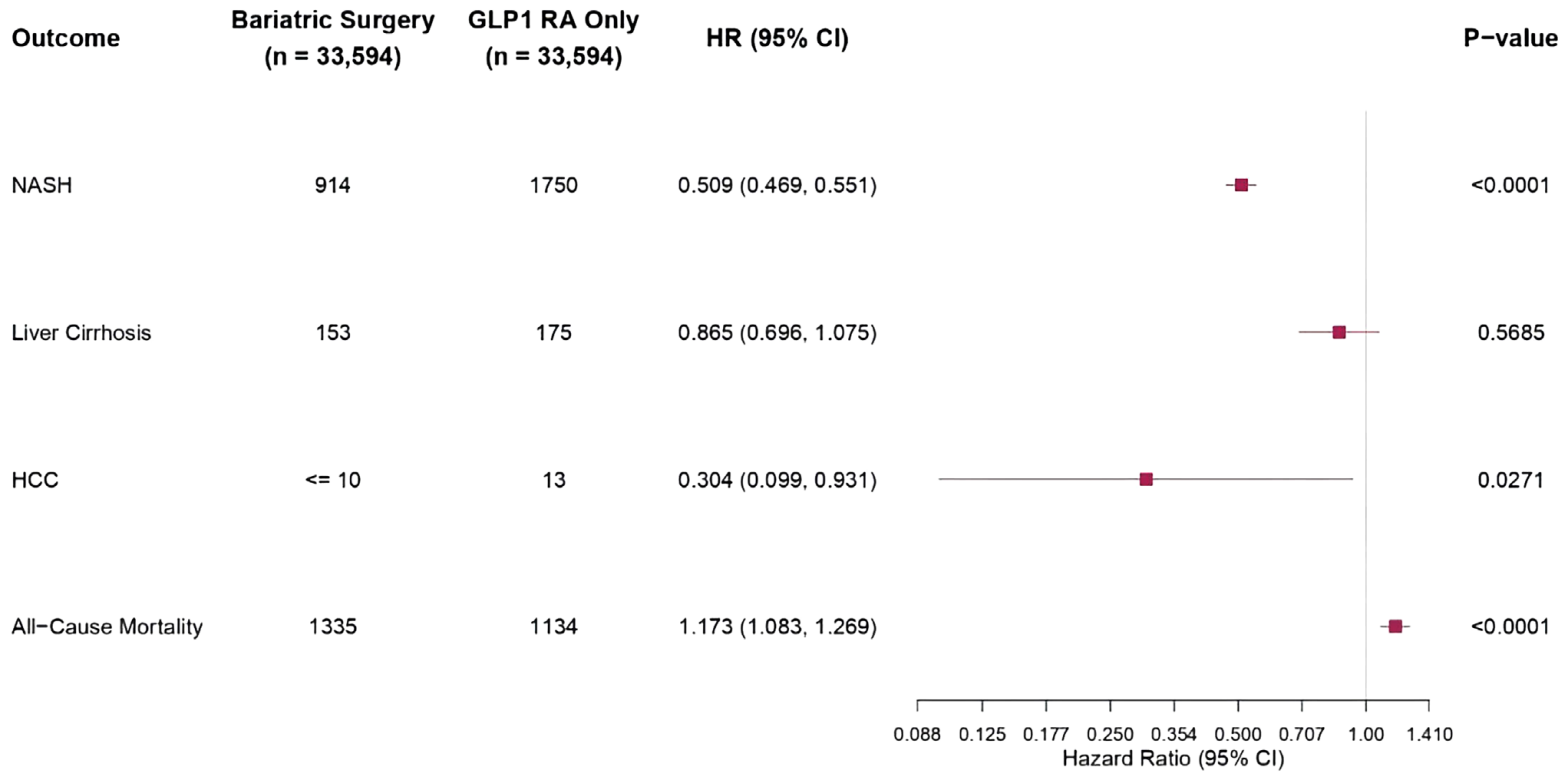
This forest plot illustrates hazard ratios (HR) and 95% confidence intervals (CI) for a variety of clinical events comparing Bariatric surgery and GLP‐1RA over 5 years post‐matching. The horizontal bars are 95% CI with significant results presented in the form of *p* values.

The total number of patients who remained in follow‐up was 33,594 patients in the BS group and 33,594 patients in the non‐BS group at the end of 1 year, 32,715 patients in the BS group and 32,715 patients in the non‐BS group at the end of 3 years, 32,512 patients in the BS group and 32,512 patients in the non‐BS group at the end of 5 years. Rates of incidence of new‐onset NASH were significantly lower in the BS group than in the matched GLP1‐RA group (Table 2 and Figure 2). Similarly, patients in the BS group had a significantly lower risk of liver cirrhosis and HCC than matched GLP1‐RA patients (Figure 2).

Similarly, the cumulative incidence of new onset of NASH at 7 years was significantly lower in the BS group (HR, 0.482; 95% CI, 0.445, 0.523) (Table S4). BS was significantly associated with a lower hazard of NASH crossing all subgroups except diabetic groups (Table S3). Kaplan–Meier survival analysis showed that the cumulative probability of being event‐free up to 7 years from the index event remained significantly lower in the non‐BS group compared with the BS group for NASH outcome (log‐rank *p* < 0.001).

### 2.6 All‐Cause Mortality

Mortality was significantly higher in the BS group than the GLP1‐RA group (HR, 0.56; 95% CI, 0.42–0.74). Kaplan‐Meier survival analyses also revealed worse survival in the BS group compared with the GLP1‐RA group (log‐rank *p* < 0.001).

### 2.7 Sensitivity Analysis

Results of the sensitivity analysis are provided in Table S3. Results of the sensitivity analysis were consistent with the results from the primary study analysis, and all statistically significant associations remained unchanged.

### 2.8 Secondary Analysis

**FIGURE 3:**
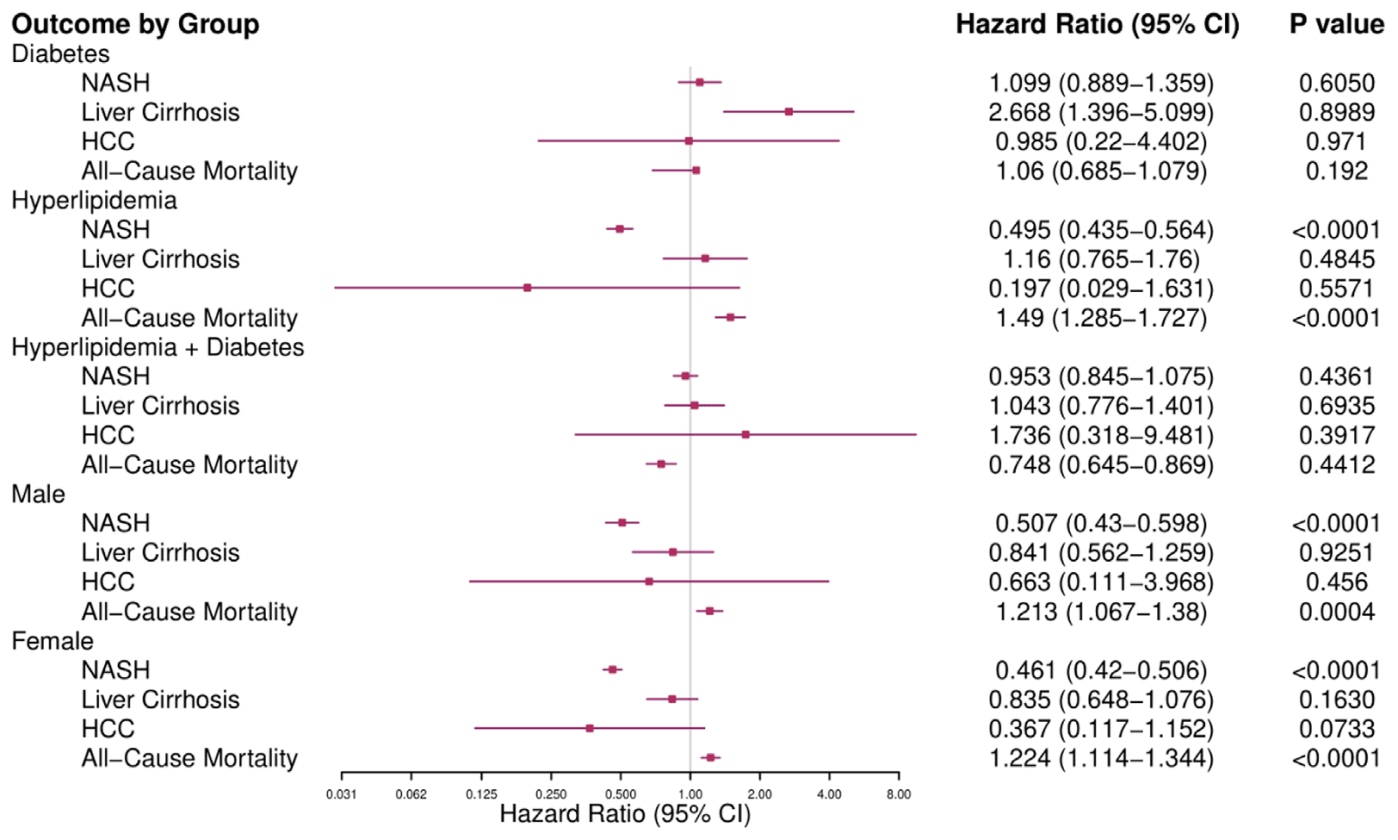
This forest plot illustrates hazard ratios (HR) and 95% confidence intervals (CI) for a variety of clinical events comparing Bariatric surgery and GLP‐1RA in diabetic, hyperlipidemia, Male and female post‐matching. The horizontal bars are the 95% CI with significant results presented in the form of *p* values.

In the secondary analysis comparing new incidence of NASH between patients with obesity male and female, Diabetes, hyperlipidemia and both diabetes and hyperlipidemia who underwent BS or GLP‐1 RA, BS was associated with a reduction in NASH outcomes, Male (HR, 0.507; 95% CI, 0.43–0.598) Female (HR, 0.461; 95% CI, 0.42–0.506) Diabetes (HR, 1.099; 95% CI, 0.889–1.359) Hyperlipidemia (HR, 0.495; 95% CI, 0.435–0.564) Hyperlipidemia and diabetes(HR, 0.953; 95% CI, 0.845–1.075) (Table S4) (Figure 3).

## 3 Discussion

NASH is a severe form of nonalcoholic fatty liver disease that involves hepatic inflammation, hepatocyte damage and fibrosis, often progressing to cirrhosis and hepatocellular carcinoma [9]. Metabolic dysfunctions, particularly obesity, insulin resistance and dyslipidemia, form the cornerstone of NASH pathogenesis, with accumulating evidence implicating genetic factors, sex hormones and inflammatory mechanisms in disease progression that may end with neoplasms [6, 10] So, the diagnosis should be made using various techniques, including non‐invasive methods such as imaging and biomarkers, or the gold‐standard invasive technique, liver biopsy, to improve detection and disease monitoring [11]. Until last year, there were no FDA‐approved drugs for treating NASH. However, the FDA recently approved resmetirom, the first drug specifically for NASH, representing a major advancement in NASH treatment [12].

Despite this breakthrough, other therapeutic options continue to be utilised even in the shade of resmetirom, GLP‐1RAs, PPAR agonists, FGF‐21 analogs and bariatric surgeries. Among these, GLP‐1 is an incretin hormone that regulates blood sugar by stimulating insulin secretion, inhibiting glucagon release and slowing gastric emptying. Its role in NASH treatment, as demonstrated by many studies such as Wood et al. [13], suggests that GLP‐1RA significantly reduces hepatic steatosis, improves liver fibrosis markers and decreases the progression of fibrosis compared to untreated patients. Additionally, Xue et al. [14] conducted a study on small‐molecule GLP‐1RA, such as cinchonine, which has shown potential in reducing hepatic fat accumulation and fibrosis in NASH models.

Similarly, bariatric surgeries, represented by Roux‐en‐Y gastric bypass and sleeve gastrectomy, serve as an effective intervention for treating NASH by substantially improving liver histology and reducing fibrosis. These procedures not only promote substantial and sustained weight loss but also induce metabolic changes that positively impact liver health, including alterations in bile acid signalling that contribute to the reversal of NASH [15]. Supporting this, Lassailly et al. [16], in their study, showed that long‐term follow‐up research indicates that up to 84% of patients achieve NASH resolution within five years after surgery. These findings, as revealed by various published studies, prompted us to conduct the first article that directly compares the role of each GLP‐1 RA and bariatric surgery in the treatment of NASH.

Our study demonstrated that BS was significantly associated with a lower incidence of new‐onset NASH compared to GLP‐1 RA therapy, with a hazard ratio (HR) of 0.509 (95% CI, 0.469–0.551). This protective effect persisted across multiple follow‐up intervals, including 1, 3, 5 and 7 years, and remained consistent in subgroup analyses, except for patients with diabetes, where no significant difference was observed. Additionally, BS was associated with a reduced risk of hepatocellular carcinoma (HR, 0.304; 95% CI, 0.099–0.931) and showed a trend toward a lower incidence of liver cirrhosis, though this did not reach statistical significance (HR, 0.865; 95% CI, 0.696–1.075). However, contrary to expectations, all‐cause mortality was higher in the BS group compared to the GLP‐1 RA cohort (HR, 1.173; 95% CI, 1.083–1.269).

In the present study, the results align with those of Wood et al., who found that GLP‐1 RAs may slow fibrosis progression in NASH. Similarly, Loomba et al. [17] showed that treatment with tirzepatide for 52 weeks was more effective than placebo in achieving the resolution of MASH without worsening fibrosis. Furthermore, Hartman et al. [18], in their randomised controlled trial, strengthened these findings by illustrating the profound effect of GLP‐1 RAs in reducing biomarkers of nonalcoholic steatohepatitis. That stresses the potential of GLP‐1 RAs not only in improving metabolic health but also in slowing liver disease progression through their anti‐inflammatory and antifibrotic effects. Syn et al. [19] conducted a meta‐analysis that revealed a lower all‐cause mortality rate in patients who underwent bariatric surgery compared to those who did not. Similarly, Dicker et al. found that bariatric surgery was associated with a 62% reduction in mortality (HR, 0.38; 95% CI, 0.25–0.58) compared to GLP‐1 RAs. This difference may be explained by the greater relative decrease in BMI observed in patients who underwent bariatric metabolic surgery (−31.4%) compared to those treated with GLP‐1 RAs (−12.8%) [20]. In contrast, our results showed a higher all‐cause mortality rate in the bariatric surgery (BS) group compared to the GLP‐1 RA group. This may be attributed to factors such as surgical complications, malnutrition and long‐term metabolic alterations, which are less common in GLP‐1 RA users.

Leveraging real‐world data, this study is the first to compare the effectiveness of BS and GLP‐1 RAs in reducing NASH and related hepatic complications. Although propensity score matching was employed to mitigate confounding, the retrospective design inherently limits causal inference. Unmeasured variables—such as lifestyle behaviours, treatment adherence and genetic predisposition—may have influenced outcomes. Furthermore, the study's reliance on North American healthcare data restricts its generalizability to broader populations. The observed increase in all‐cause mortality among BS recipients raises concerns that merit further investigation to determine whether the cause lies in procedural risks, patient selection bias or residual confounding.

Importantly, the analysis was limited to GLP‐1 RAs available during the data collection period (2014–2019), chiefly liraglutide and semaglutide. Newer agents like tirzepatide—introduced after 2020 and characterised by dual GIP and GLP‐1 receptor agonist activity—may demonstrate different clinical effects. As such, these findings should not be generalised to newer or mechanistically distinct anti‐obesity therapies without additional research. Prospective trials with long‐term follow‐up are essential to confirm these results and elucidate the mechanisms underlying observed differences.

## 4 Conclusion

This study shows that bariatric surgery is more effective than GLP‐1 receptor agonists in reducing the risk of NASH, liver cirrhosis and hepatocellular carcinoma in obese patients. These benefits were consistent over time. However, the higher mortality rate in the bariatric surgery group raises concerns that need further research. Future studies should explore the reasons behind this and evaluate the best treatment approaches for long‐term health.

## Author Contributions

Abdallah Hussein led the study design, analysis and manuscript drafting. Ameer Awashra contributed to data analysis, supervision and critical revisions. Islam Rajab supported software and data validation. Mohammad Bdair and Dawoud Hamdan assisted with data collection and editing. Ahmad Nouri and Elaf Khatib contributed to methodology, literature review and figures. Ghiras Khatib managed references and reviewed the manuscript. Nyan Latt supervised the project and approved the final version. All authors reviewed and approved the manuscript.

## Ethics Statement

This retrospective study is exempt from informed consent. The data reviewed is a secondary analysis of existing data, does not involve intervention or interaction with human subjects, and is de‐identified per the de‐identification standard defined in Section §164.514(a) of the HIPAA Privacy Rule. The process by which the data is de‐identified is attested to through a formal determination by a qualified expert as defined in Section §164.514(b)(1) of the HIPAA Privacy Rule. This formal determination by a qualified expert was refreshed in December 2020.

## Conflicts of Interest

The authors declare no conflicts of interest.

## Supporting information


Data S1.

## Data Availability

The data that support the findings of this study are available on request from the corresponding author. The data are not publicly available due to privacy or ethical restrictions.
