# Supplementary material for: Comparative Effectiveness of Bariatric Surgery Versus GLP‐1 Receptor Agonists in Reducing the Risk of New‐Onset of NASH: A Retrospective Multinational Cohort Study From North America and Europe
Source: Endocrinol Diabetes Metab. 2025 Jul 12;8(4):e70075. doi: 10.1002/edm2.70075 (PMC12255231; doi:10.1002/edm2.70075)
Supplement: Supplementary file 1 — Data S1. [file EDM2-8-e70075-s001.docx]

eTable1 Demographic, Diagnostic, Procedural, Medication, Visit, and Laboratory Codes Used in the Definition of the Cohorts.

eTable2 Diagnostic, Visit, and Procedural Codes Used in the Definition of Outcomes.

eTable3 Sensitivity Analysis Measuring Outcomes Associated with Bariatric Surgery vs GLP-1RA after Excluding Index Events Within 2 Years After the Index Date.

eTable 4 Secondary analysis measuring Outcomes Associated with Bariatric Surgery vs GLP-1RA in Diabetes, Hyperlipidemia, male and female groups.

| eTable1 Demographic, Diagnostic, Procedural, Medication, Visit, and Laboratory Codes Used in the Definition of the Cohorts | | | | | |
| --- | --- | --- | --- | --- | --- |
| Bariatric surgery | | | | | |
|  | must have |  | demographics | Age | Age (at least 18 years (most recent occurrence)) |
| Group 1 | | | | | |
|  | **Group 1A** | | | | |
|  | must have | any of | laboratory | TNX:9083 | BMI (at least 30.00 kg/m2) |
|  |  |  | diagnosis | UMLS:ICD10CM:E66.9 | Obesity, unspecified |
|  |  |  | diagnosis | UMLS:ICD10CM:E66.0 | Obesity due to excess calories |
|  |  |  | diagnosis | UMLS:ICD10CM:E66.81 | Obesity class |
|  |  |  | diagnosis | UMLS:ICD10CM:E66.3 | Overweight |
|  |  |  | diagnosis | UMLS:ICD10CM:E65-E68 | Overweight, obesity and other hyperalimentation |
|  |  |  | diagnosis | UMLS:ICD10CM:E66 | Overweight and obesity |
|  | date constraint | | The terms in this group occurred on or before Dec 31, 2019 | | |
|  | event relationship | | Any instance of Group 1B occurred on or before any instance of Group 1A | | |
|  | **Group 1B** | | | | |
|  | cannot have |  | diagnosis | UMLS:ICD10CM:K74.60 | Unspecified cirrhosis of liver |
|  |  | or | diagnosis | UMLS:ICD10CM:K70.30 | Alcoholic cirrhosis of liver without ascites |
|  |  | or | diagnosis | UMLS:ICD10CM:K70.3 | Alcoholic cirrhosis of liver |
|  |  | or | diagnosis | UMLS:ICD10CM:K76.6 | Portal hypertension |
|  |  | or | diagnosis | UMLS:ICD10CM:B18.2 | Chronic viral hepatitis C |
|  |  | or | diagnosis | UMLS:ICD10CM:K70.31 | Alcoholic cirrhosis of liver with ascites |
|  |  | or | diagnosis | UMLS:ICD10CM:K74.69 | Other cirrhosis of liver |
|  |  | or | diagnosis | UMLS:ICD10CM:K74.6 | Other and unspecified cirrhosis of liver |
|  |  | or | diagnosis | UMLS:ICD10CM:K72.90 | Hepatic failure, unspecified without coma |
|  |  | or | diagnosis | UMLS:ICD10CM:K72.10 | Chronic hepatic failure without coma |
|  |  | or | diagnosis | UMLS:ICD10CM:G93.41 | Metabolic encephalopathy |
|  |  | or | diagnosis | UMLS:ICD10CM:K76.82 | Hepatic encephalopathy |
|  |  | or | diagnosis | UMLS:ICD10CM:B19.9 | Unspecified viral hepatitis without hepatic coma |
|  |  | or | diagnosis | UMLS:ICD10CM:E08-E13 | Diabetes mellitus |
|  |  | or | diagnosis | UMLS:ICD10CM:E11 | Type 2 diabetes mellitus |
|  |  | or | diagnosis | UMLS:ICD10CM:K75.81 | Nonalcoholic steatohepatitis (NASH) |
|  |  | or | diagnosis | UMLS:ICD10CM:K76.0 | Fatty (change of) liver, not elsewhere classified |
|  |  | or | diagnosis | UMLS:ICD10CM:C22.0 | Liver cell carcinoma |
|  |  | or | diagnosis | UMLS:ICD10CM:C22.8 | Malignant neoplasm of liver, primary, unspecified as to type |
|  |  | or | diagnosis | UMLS:ICD10CM:C22.2 | Hepatoblastoma |
|  |  | or | diagnosis | UMLS:ICD10CM:C22.7 | Other specified carcinomas of liver |
|  |  | or | diagnosis | UMLS:ICD10CM:B15-B19 | Viral hepatitis |
|  |  | or | diagnosis | UMLS:ICD10CM:B19 | Unspecified viral hepatitis |
|  |  | or | diagnosis | UMLS:ICD10CM:B19.10 | Unspecified viral hepatitis B without hepatic coma |
|  |  | or | diagnosis | UMLS:ICD10CM:B19.20 | Unspecified viral hepatitis C without hepatic coma |
|  |  | or | diagnosis | UMLS:ICD10CM:B18 | Chronic viral hepatitis |
|  |  | or | diagnosis | UMLS:ICD10CM:B17.8 | Other specified acute viral hepatitis |
| Group 2 | | | | | |
|  | **Group 2A** | | | | |
|  | must have | any of | laboratory | TNX:9083 | BMI (at least 30.00 kg/m2) |
|  |  |  | diagnosis | UMLS:ICD10CM:E66.9 | Obesity, unspecified |
|  |  |  | diagnosis | UMLS:ICD10CM:E66.0 | Obesity due to excess calories |
|  |  |  | diagnosis | UMLS:ICD10CM:E66.81 | Obesity class |
|  |  |  | diagnosis | UMLS:ICD10CM:E66.3 | Overweight |
|  |  |  | diagnosis | UMLS:ICD10CM:E65-E68 | Overweight, obesity and other hyperalimentation |
|  |  |  | diagnosis | UMLS:ICD10CM:E66 | Overweight and obesity |
|  | date constraint | | The terms in this group occurred on or before Dec 31, 2019 | | |
|  | event relationship | | Any instance of Group 2B occurred within 1 year on or after any instance of Group 2A | | |
|  | **Group 2B** | | | | |
|  | must have | any of | procedure | UMLS:CPT:43842 | Gastric restrictive procedure, without gastric bypass, for morbid obesity; vertical-banded gastroplasty |
|  |  |  | procedure | UMLS:CPT:43775 | Laparoscopy, surgical, gastric restrictive procedure; longitudinal gastrectomy (ie, sleeve gastrectomy) |
|  |  |  | procedure | UMLS:CPT:43845 | Gastric restrictive procedure with partial gastrectomy, pylorus-preserving duodenoileostomy and ileoileostomy (50 to 100 cm common channel) to limit absorption (biliopancreatic diversion with duodenal switch) |
|  |  |  | procedure | UMLS:CPT:43846 | Gastric restrictive procedure, with gastric bypass for morbid obesity; with short limb (150 cm or less) Roux-en-Y gastroenterostomy |
|  |  |  | procedure | UMLS:SNOMED:430715008 | Bariatric operative procedure |
|  |  |  | procedure | UMLS:CPT:43644 | Laparoscopy, surgical, gastric restrictive procedure; with gastric bypass and Roux-en-Y gastroenterostomy (roux limb 150 cm or less) |
|  |  |  | procedure | UMLS:CPT:43770 | Laparoscopy, surgical, gastric restrictive procedure; placement of adjustable gastric restrictive device (eg, gastric band and subcutaneous port components) |
|  |  |  | procedure | UMLS:CPT:1007386 | Laparoscopic Bariatric Surgery Procedures |
|  |  |  | procedure | UMLS:CPT:1007385 | Bariatric Surgery Procedures |
|  |  |  | procedure | UMLS:CPT:43645 | Laparoscopy, surgical, gastric restrictive procedure; with gastric bypass and small intestine reconstruction to limit absorption |
|  |  |  | diagnosis | UMLS:ICD10CM:Z98.84 | Bariatric surgery status |
|  | cannot have |  | medication | NLM:ATC:A10BK | Sodium-glucose co-transporter 2 (SGLT2) inhibitors |
|  |  | or | medication | NLM:RXNORM:2601723 | tirzepatide |
|  |  | or | medication | NLM:ATC:A10BJ | Glucagon-like peptide-1 (GLP-1) analogues |

| GLP-1RA group | | | | | |
| --- | --- | --- | --- | --- | --- |
|  | must have |  | demographics | Age | Age (at least 18 years (most recent occurrence)) |
| Group 1 | | | | | |
|  | **Group 1A** | | | | |
|  | must have | any of | laboratory | TNX:9083 | BMI (at least 30.00 kg/m2) |
|  |  |  | diagnosis | UMLS:ICD10CM:E66.9 | Obesity, unspecified |
|  |  |  | diagnosis | UMLS:ICD10CM:E66.0 | Obesity due to excess calories |
|  |  |  | diagnosis | UMLS:ICD10CM:E66.81 | Obesity class |
|  |  |  | diagnosis | UMLS:ICD10CM:E66.3 | Overweight |
|  |  |  | diagnosis | UMLS:ICD10CM:E65-E68 | Overweight, obesity and other hyperalimentation |
|  |  |  | diagnosis | UMLS:ICD10CM:E66 | Overweight and obesity |
|  | date constraint | | The terms in this group occurred on or before Dec 31, 2019 | | |
|  | event relationship | | Any instance of Group 1B occurred on or before any instance of Group 1A | | |
|  | **Group 1B** | | | | |
|  | cannot have |  | diagnosis | UMLS:ICD10CM:K74.60 | Unspecified cirrhosis of liver |
|  |  | or | diagnosis | UMLS:ICD10CM:K70.30 | Alcoholic cirrhosis of liver without ascites |
|  |  | or | diagnosis | UMLS:ICD10CM:K70.3 | Alcoholic cirrhosis of liver |
|  |  | or | diagnosis | UMLS:ICD10CM:K76.6 | Portal hypertension |
|  |  | or | diagnosis | UMLS:ICD10CM:B18.2 | Chronic viral hepatitis C |
|  |  | or | diagnosis | UMLS:ICD10CM:K70.31 | Alcoholic cirrhosis of liver with ascites |
|  |  | or | diagnosis | UMLS:ICD10CM:K74.69 | Other cirrhosis of liver |
|  |  | or | diagnosis | UMLS:ICD10CM:K74.6 | Other and unspecified cirrhosis of liver |
|  |  | or | diagnosis | UMLS:ICD10CM:K72.90 | Hepatic failure, unspecified without coma |
|  |  | or | diagnosis | UMLS:ICD10CM:K72.10 | Chronic hepatic failure without coma |
|  |  | or | diagnosis | UMLS:ICD10CM:G93.41 | Metabolic encephalopathy |
|  |  | or | diagnosis | UMLS:ICD10CM:K76.82 | Hepatic encephalopathy |
|  |  | or | diagnosis | UMLS:ICD10CM:B19.9 | Unspecified viral hepatitis without hepatic coma |
|  |  | or | diagnosis | UMLS:ICD10CM:E08-E13 | Diabetes mellitus |
|  |  | or | diagnosis | UMLS:ICD10CM:E11 | Type 2 diabetes mellitus |
|  |  | or | diagnosis | UMLS:ICD10CM:K75.81 | Nonalcoholic steatohepatitis (NASH) |
|  |  | or | diagnosis | UMLS:ICD10CM:K76.0 | Fatty (change of) liver, not elsewhere classified |
|  |  | or | diagnosis | UMLS:ICD10CM:C22.0 | Liver cell carcinoma |
|  |  | or | diagnosis | UMLS:ICD10CM:C22.8 | Malignant neoplasm of liver, primary, unspecified as to type |
|  |  | or | diagnosis | UMLS:ICD10CM:C22.2 | Hepatoblastoma |
|  |  | or | diagnosis | UMLS:ICD10CM:C22.7 | Other specified carcinomas of liver |
|  |  | or | diagnosis | UMLS:ICD10CM:B15-B19 | Viral hepatitis |
|  |  | or | diagnosis | UMLS:ICD10CM:B19 | Unspecified viral hepatitis |
|  |  | or | diagnosis | UMLS:ICD10CM:B19.10 | Unspecified viral hepatitis B without hepatic coma |
|  |  | or | diagnosis | UMLS:ICD10CM:B19.20 | Unspecified viral hepatitis C without hepatic coma |
|  |  | or | diagnosis | UMLS:ICD10CM:B18 | Chronic viral hepatitis |
|  |  | or | diagnosis | UMLS:ICD10CM:B17.8 | Other specified acute viral hepatitis |
| Group 2 | | | | | |
|  | **Group 2A** | | | | |
|  | must have | any of | laboratory | TNX:9083 | BMI (at least 30.00 kg/m2) |
|  |  |  | diagnosis | UMLS:ICD10CM:E66.9 | Obesity, unspecified |
|  |  |  | diagnosis | UMLS:ICD10CM:E66.0 | Obesity due to excess calories |
|  |  |  | diagnosis | UMLS:ICD10CM:E66.81 | Obesity class |
|  |  |  | diagnosis | UMLS:ICD10CM:E66.3 | Overweight |
|  |  |  | diagnosis | UMLS:ICD10CM:E65-E68 | Overweight, obesity and other hyperalimentation |
|  |  |  | diagnosis | UMLS:ICD10CM:E66 | Overweight and obesity |
|  | date constraint | | The terms in this group occurred on or before Dec 31, 2019 | | |
|  | event relationship | | Any instance of Group 2B occurred within 1 year on or after any instance of Group 2A | | |
|  | **Group 2B** | | | | |
|  | must have |  | medication | NLM:ATC:A10BJ | Glucagon-like peptide-1 (GLP-1) analogues |
|  | cannot have |  | medication | NLM:ATC:A10BK | Sodium-glucose co-transporter 2 (SGLT2) inhibitors |
|  |  | or | medication | NLM:RXNORM:2601723 | tirzepatide |
|  |  | or | procedure | UMLS:CPT:43842 | Gastric restrictive procedure, without gastric bypass, for morbid obesity; vertical-banded gastroplasty |
|  |  | or | procedure | UMLS:CPT:43846 | Gastric restrictive procedure, with gastric bypass for morbid obesity; with short limb (150 cm or less) Roux-en-Y gastroenterostomy |
|  |  | or | procedure | UMLS:SNOMED:430715008 | Bariatric operative procedure |
|  |  | or | diagnosis | UMLS:ICD10CM:Z98.84 | Bariatric surgery status |
|  |  | or | procedure | UMLS:CPT:1007385 | Bariatric Surgery Procedures |
|  |  | or | procedure | UMLS:CPT:1007386 | Laparoscopic Bariatric Surgery Procedures |
|  |  | or | procedure | UMLS:CPT:43770 | Laparoscopy, surgical, gastric restrictive procedure; placement of adjustable gastric restrictive device (eg, gastric band and subcutaneous port components) |
|  |  | or | procedure | UMLS:CPT:43644 | Laparoscopy, surgical, gastric restrictive procedure; with gastric bypass and Roux-en-Y gastroenterostomy (roux limb 150 cm or less) |
|  |  | or | procedure | UMLS:CPT:43775 | Laparoscopy, surgical, gastric restrictive procedure; longitudinal gastrectomy (ie, sleeve gastrectomy) |
|  |  | or | procedure | UMLS:CPT:43645 | Laparoscopy, surgical, gastric restrictive procedure; with gastric bypass and small intestine reconstruction to limit absorption |
|  |  | or | procedure | UMLS:CPT:43845 | Gastric restrictive procedure with partial gastrectomy, pylorus-preserving duodenoileostomy and ileoileostomy (50 to 100 cm common channel) to limit absorption (biliopancreatic diversion with duodenal switch) |

**eTable2** Diagnostic, Visit, and Procedural Codes Used in the Definition of Outcomes

| Nonalcoholic steatohepatitis NASH | | | | |
| --- | --- | --- | --- | --- |
|  | Outcome definition | | | |
|  | | Diagnosis | UMLS:ICD10CM:K76.0 | Fatty (change of) liver, not elsewhere classified |
|  | | Diagnosis | UMLS:ICD10CM:K75.81 | Nonalcoholic steatohepatitis (NASH) |
|  | | Diagnosis | UMLS:ICD10CM:K74 | Fibrosis and cirrhosis of liver |
|  | | Laboratory | UMLS:LNC:48795-9 | Fibrosis score (at least 1.00 {score} (most recent occurrence)) |
| Mortality | | | | |
|  | Outcome definition | | | |
|  | | Demographics | Deceased | Deceased |
|  | | Diagnosis | UMLS:ICD10CM:R99 | Ill-defined and unknown cause of mortality |
|  | | Diagnosis | UMLS:ICD10CM:R99-R99 | Ill-defined and unknown cause of mortality (R99) |
|  | | Diagnosis | UMLS:ICD10CM:R69 | Illness, unspecified |
| Liver Cirrhosis | | | | |
|  | Outcome definition | | | |
|  | | Diagnosis | UMLS:ICD10CM:K74.0 | Hepatic fibrosis |
|  | | Diagnosis | UMLS:ICD10CM:K74.60 | Unspecified cirrhosis of liver |
|  | | Diagnosis | UMLS:ICD10CM:K74 | Fibrosis and cirrhosis of liver |
|  | | Diagnosis | UMLS:ICD10CM:K74.6 | Other and unspecified cirrhosis of liver |
| Hepatocellular carcinoma | | | | |
|  | Outcome definition | | | |
|  | | GlobalOncology | UMLS:ICDO3:8171/3 | Hepatocellular carcinoma, fibrolamellar |
|  | | GlobalOncology | UMLS:ICDO3:8170/3 | Hepatocellular carcinoma, NOS |
|  | | GlobalOncology | UMLS:ICDO3:8174/3 | Hepatocellular carcinoma, clear cell type |
|  | | Diagnosis | UMLS:ICD10CM:C22.0 | Liver cell carcinoma |
|  | | Diagnosis | UMLS:ICD10CM:C22.8 | Malignant neoplasm of liver, primary, unspecified as to type |
|  | | Diagnosis | UMLS:ICD10CM:C22.7 | Other specified carcinomas of liver |

| eTable 3: Sensitivity Analysis Measuring Outcomes Associated With Bariatric Surgery vs GLP-1RA after Excluding Index Events Within 2 Years After the Index Date | | | | | |
| --- | --- | --- | --- | --- | --- |
| At 7 years (after excluding a year from the index date) | | | | | |
| Outcome | Bariatric surgery (n = 32929) | GLP1 RA Only (n = 32929) | Hazard Ratio (HR) | 95% Confidence Interval (CI) | P-Value |
| NASH | 904 | 1729 | 0.482 | (0.445,0.523) | <0.0001 |
| Liver Cirrhosis | 1169 | 178 | 0.891 | (0.722,1.1) | 0.2853 |
| HCC | ≤ 10 | 14 | 0.269 | (0.088,0.818) | 0.0131 |
| All-Cause Mortality | 1083 | 949 | 1.087 | (0.996,1.186) | <0.0609 |
| At 7 years (after excluding 2 years from the index date) | | | | | |
| Outcome | Bariatric surgery (n = **34,234**) | GLP1 RA Only (n = **34,234**) | Hazard Ratio (HR) | 95% Confidence Interval (CI) | P-Value |
| NASH | 828 | 1580 | 0.483 | (0.444,0.525) | <0.0001 |
| Liver Cirrhosis | 145 | 159 | 0.842 | (0.672,1.055) | 0.1355 |
| HCC | ≤ 10* | 12 | 0.467 | 0.175,1.245 | 0.1192 |
| All-Cause Mortality | 888 | 760 | 1.093 | (0.992,1.205) | 0.0711 |

| eTable 4 Secondary analysis measuring Outcomes Associated with Bariatric Surgery vs GLP-1RA in Diabetes, Hyperlipidemia, male and female groups. | | | | | | |
| --- | --- | --- | --- | --- | --- | --- |
|  | **Outcome** | **Bariatric surgery** | **GLP1 RA Only** | **Hazard Ratio (HR)** | **95% Confidence Interval (CI)** | **P-Value** |
|  | | Total N. 3182 | Total N. 3182 |  | | |
| Diabetes | NASH | 202 | 148 | 1.099 | (0.889,1.359) | 0.6050 |
|  | Liver Cirrhosis | 39 | 12 | 2.668 | (1.396,5.099) | 0.8989 |
|  | HCC | ≤ 10 | ≤ 10 | 0.985 | (0.22,4.402) | 0.971 |
|  | All-Cause Mortality | 154 | 145 | 1.06 | (0.685,1.079) | 0.192 |
|  | | Total N. 10602 | Total N 10602 |  | | |
| Hyperlipidemia | NASH | 341 | 670 | 0.495 | (0.435, 0.564) | <0.0001 |
|  | Liver Cirrhosis | 48 | 41 | 1.16 | (0.765,1.76) | 0.4845 |
|  | HCC | ≤ 10 | ≤ 10 | 0.197 | (0.029,1.631) | 0.5571 |
|  | All-Cause Mortality | 437 | 293 | 1.49 | (1.285,1.727) | <0.0001 |
|  |  | Total N. 6459 | Total N. 6459 |  |  |  |
| Hyperlipidemia+ Diabetes | NASH | 549 | 517 | 0.953 | (0.845,1.075) | 0.4361 |
|  | Liver Cirrhosis | 95 | 82 | 1.043 | (0.776,1.401) | 0.6935 |
|  | HCC | ≤ 10 | ≤ 10 | 1.736 | (0.318,9.481) | 0.3917 |
|  | All-Cause Mortality | 315 | 382 | 0.748 | (0.645,0.869) | 0.4412 |
|  |  | Total N.8850 | Total N. 8850 |  |  |  |
| Male | NASH | 215 | 411 | 0.507 | (0.43,0.598) | <0.0001 |
|  | Liver Cirrhosis | 44 | 51 | 0.841 | (0.562,1.259) | 0.9251 |
|  | HCC | ≤ 10 | ≤ 10 | 0.663 | (0.111,3.968) | 0.456 |
|  | All-Cause Mortality | 514 | 423 | 1.213 | (1.067,1.38) | 0.0004 |
|  |  | Total N. 26349 | Total N. 26349 |  |  |  |
| Female | NASH | 637 | 1371 | 0.461 | (0.42,0.506) | <0.0001 |
|  | Liver Cirrhosis | 109 | 132 | 0.835 | (0.648,1.076) | 0.1630 |
|  | HCC | ≤ 10 | 11 | 0.367 | (0.117,1.152) | 0.0733 |
|  | All-Cause Mortality | 957 | 794 | 1.224 | (1.114,1.344) | <0.0001 |
